# Centrosome amplification induced by survivin suppression enhances both chromosome instability and radiosensitivity in glioma cells

**DOI:** 10.1038/sj.bjc.6604160

**Published:** 2008-01-15

**Authors:** T Saito, S Hama, H Izumi, F Yamasaki, Y Kajiwara, S Matsuura, K Morishima, T Hidaka, P Shrestha, K Sugiyama, K Kurisu

**Affiliations:** 1 Department of Neurosurgery, Graduate School of Biomedical Sciences, Hiroshima University, 1-2-3 Kasumi, Minami-ku, Hiroshima 734-8551, Japan; 2 Department of Radiation Biology, Research Institute for Radiation Biology and Medicine, Hiroshima University, 1-2-3 Kasumi, Minami-ku, Hiroshima 734-8553, Japan

**Keywords:** malignant glioma, survivin, chromosome instability, radiosensitivity, centrosome

## Abstract

Glioblastoma is characterised by invasive growth and a high degree of radioresistance. Survivin, a regulator of chromosome segregation, is highly expressed and known to induce radioresistance in human gliomas. In this study, we examined the effect of survivin suppression on radiosensitivity in malignant glioma cells, while focusing on centrosome aberration and chromosome instability (CIN). We suppressed survivin by small interfering RNA transfection, and examined the radiosensitivity using a clonogenic assay and a trypan blue exclusion assay in U251MG (p53 mutant) and D54MG (p53 wild type) cells. To assess the CIN status, we determined the number of centrosomes using an immunofluorescence analysis, and the centromeric copy number by fluorescence *in situ* hybridisation. As a result, the radiosensitisation differed regarding the p53 status as U251MG cells quickly developed extreme centrosome amplification (=CIN) and enhanced the radiosensitivity, while centrosome amplification and radiosensitivity increased more gradually in D54MG cells. TUNEL assay showed that survivin inhibition did not lead to apoptosis after irradiation. This cell death was accompanied by an increased degree of aneuploidy, suggesting mitotic cell death. Therefore, survivin inhibition may be an attractive therapeutic target to overcome the radioresistance while, in addition, proper attention to CIN (centrosome number) is considered important for improving radiosensitivity in human glioma.

Human malignant gliomas diffusely invade the surrounding normal brain tissue. The surgical removal of the entire tumour is thus frequently difficult, and radiation therapy is often administered as an adjuvant in the treatment of incompletely resected tumours. However, the clinical effectiveness of irradiation has been limited. In almost all cases, tumours are refractory to current treatments, and such patients normally die from brain herniation due to unrestrained growth of the tumour (Giangaspero and Burger, 1983). The median survival time of malignant glioma patients is less than 2 years, despite extensive and multidisciplinary treatment.

Survivin has multiple functions. It inhibits apoptosis (Altieri, 2001) and is involved in the spindle checkpoint and the regulation of proper chromosomal segregation during mitosis by acting as a member of the chromosomal passenger protein family (Li *et al*, 1998; Adams *et al*, 2001). A number of studies have demonstrated that the suppression of survivin results in cells with multipolar mitotic spindles that are either multinucleated or aneuploid (Li *et al*, 1999; Beltrami *et al*, 2004; Kappler *et al*, 2004). Some studies have also indicated that aneuploidy promotes chromosome instability (CIN) in glioma and cervical carcinoma cells (Nitta *et al*, 2002; Olaharski *et al*, 2006).

Chromosome instability generally results from mitotic defects, and it promotes tumour progression. Therefore, CIN was thought to be a clinical malignant feature in almost all types of malignant tumours. A number of studies have shown that centrosome amplification contributes to CIN in tumour cells (D'Assoro *et al*, 2002; Doxsey, 2002; Kawamura *et al*, 2004). The centrosome is a major microtubule-organising centre in animal cells (Doxsey, 2001). During mitosis, centrosomes regulate the formation of bipolar mitotic spindles, which is an essential event for accurate chromosome segregation (Fukasawa, 2002). Because each daughter cell receives only one centrosome during cytokinesis, the centrosome must therefore duplicate once before mitosis, which normally occurs at the time of S-phase entry (Mazia, 1987). Therefore, a cell contains either one unduplicated centrosome or two duplicated centrosomes, and the numeral homoeostasis of centrosomes is thus strictly controlled. When this control is dysregulated, then centrosome amplification occurs, thus leading to aberrant mitotic spindle formation and an increased frequency of chromosome segregation errors (Doxsey, 2001; Fukasawa, 2002). It was therefore suggested that mitotic error induced by the suppression of survivin could thus lead to centrosome aberration. Previously, Li *et al* (1999) reported that interference with survivin caused a pleiotropic cell-division defect, with such features as supernumerary centrosomes, aberrant mitotic spindles, multinucleation, and polyploidy. As a result, the mitotic error induced by the suppression of survivin was thus suggested to lead to the development of a centrosome aberration and CIN.

In clinical samples, a high expression of survivin in malignant tumour cells has been reported to correlate with a shorter survival (Ferrandina *et al*, 2005; Lee *et al*, 2005; Atikcan *et al*, 2006; Sohn *et al*, 2006) and we also previously showed the survivin expression to be a significant prognostic marker for malignant gliomas (Kajiwara *et al*, 2003; Saito *et al*, 2007). Furthermore, survivin is also involved in radioresistance (Asanuma *et al*, 2002; Chakravarti *et al*, 2004; Rodel *et al*, 2005) and its suppression leads to an enhanced radiosensitivity in various malignant tumour cells (Pennati *et al*, 2003; Lu *et al*, 2004; Kappler *et al*, 2005). However, the mechanisms underlying radiosensitisation are still not well understood.

The suppression of survivin is thought to enhance radiosensitivity, while it could also lead to the induction of CIN. From these observations, the suppression of survivin is thought to include contradictory features that CIN reflects a malignant phenotype (clinical malignancy), but it enhances radiosensitivity (clinical benefit). Only one previous report examined the association between centrosome amplification and survivin (Li *et al*, 1999), while this report did not examine the effect on the radiosensitivity. Therefore, we focused our attention on the centrosome aberration leading to CIN, and the effect on the radiosensitivity induced by survivin downregulation in malignant glioma cell lines (both p53 mutant and wild type). As a result, we herein demonstrate, for the first time, that the degree of centrosome amplification correlated well with the radiosensitisation associated with the p53 status.

## MATERIALS AND METHODS

### Cell lines and cell culture

U251MG cells (human glioma; p53 mutated at codon 273 (CGT/CAT Arg/His)) and D54MG cells (human glioma; p53 wild type) were obtained in the same manner as previously described (Hama *et al*, 2003).

### Irradiation and siRNA transfection

Small interfering RNA (siRNA) against survivin sequence 5′-GCAUUCGUCCGGUUGCGCUtt-3′, antisense 5′-GGACCACCGCAUCUCUACAtt-3′, siRNA against p53 sequence (see Supplementary Information) (sc-29435; Santa Cruz Biotech, Santa Cruz, CA, USA), and a scrambled nonsense control (Ambion, Austin, TX, USA) were used for transfection. A total of 1–10 × 10^4^ cells per well were incubated in six-well plates (Falcon; Becton Dickinson, Lincoln Park, NJ, USA) 24 h before transfection; the confluency of the cell monolayer at the time of transfection was 50–70%. The cells were cultured in serum-free OptiMEM medium (Invitrogen, Karlsruhe, Germany) for 1 h before the start of transfection. The siRNA/survivin or siRNA/control was transfected with 50 nM siRNA duplexes using the HVJ Envelope VECTOR KIT (Ishihara Sangyo, Osaka, Japan) according to the manufacturer's instructions, and incubated for 4 h followed by medium change with fresh MEM. After a further 20 h incubation, the cells were irradiated in six-well plates with serum at room temperature using a ^60^Co source at a dose rate of 0.65 Gy min^−1^. The field strength of the Cs-137 was obtained using the tertiary standard Japanese Association of Radiological Physicist (JARP) ionisation chamber (accuracy is ⩽2% and repeatability is 0.5%).

### Immunoblot analysis

On days 1 (24 h), 3 (72 h), and 5 (120 h) after transfection with siRNA/control or survivin, cell lysates from U251MG (mutant p53 (mt-p53)) and D54MG (wild-type p53 (wt-p53)) were extracted using 100 *μ*l ice-cold lysis buffer (25 mM Tris-HCl, pH 7.4, 50 mM NaCl, 0.5% Na deoxycholate, 2% Nonidet P-40, 0.2% SDS, 1 mM phenylmethylsulphonyl fluoride and 50 *μ*g ml^−1^ aprotinin). The lysates were boiled, size-fractionated through 15% SDS—PAGE, and then were transferred onto a polyvinylidene-difluoride membrane (Millipore, Bedford, MA, USA). The membrane was blocked with TBS containing 0.05% Tween 20 and 5% skimmed milk and then was probed with anti-human-survivin rabbit polyclonal antibody (sc-10811; Santa Cruz Biotech), anti-p53 mouse polyclonal antibody (sc-98; Santa Cruz Biotech) and antiactin mouse monoclonal antibody (sc-1616; Santa Cruz Biotech), and finally was enhanced with chemiluminescence by ECL western blotting detection reagents (Amersham, Arlington Heights, IL, USA).

### Cell viability and cell survival analysis

Cell survival was assessed using clonogenic assays in a monolayer culture, as we have described previously (Hama *et al*, 2003). U251MG and D54MG cells were transfected with siRNA/control or survivin and, after incubation for 24 h, they were irradiated in six-well plates (Falcon) with 2, 4, or 6 Gy. The plates were returned to the incubator and allowed to proliferate in fresh medium for 14 days. When the colonies reached 50–100 cells, they were counted by staining with 0.1% crystal violet in 0.9% saline for 30 min at room temperature. The surviving fractions were calculated relative to the survival of nonirradiated nontransfected cells. These experiments were repeated twice.

The viability of cells with or without irradiation (4 Gy) was analysed using trypan blue exclusion, as previously described (Hama *et al*, 2003). Trypan blue staining was used to determine the total cell counts and viable cells on days 1 (24 h), 3 (72 h), and 5 (120 h) after siRNA transfection or irradiation. Floating and adherent cells were collected at the indicated times, were sedimented by centrifugation, and then were resuspended in MEM. The cells were thereafter diluted 1 : 9 with 0.4% trypan blue (Sigma, St Louis, MO, USA) and scored under light microscopy. Viable (unstained) and nonviable (blue-stained) cells were counted and the total number of living and dead cells were thus calculated. The results are presented as the mean±s.d., with a minimum of 500 cells being scored. Each experiment was repeated at least twice.

### Analysis of the cell cycle by flow cytometry

The cell-cycle status was analysed using flow cytometry. After siRNA/survivin or control transfection with/without irradiation (4 Gy), the cells in six-well plates were collected by trypsinisation after 24, 72, and 120 h incubation. We did not collect any floating cells to exclude dead cells and debris. The collected cells were washed twice with phosphate-buffered saline (PBS), fixed with 75% ethanol, and stored at 4°C for 48 h. After centrifugation at 400 **g** for 10 min in a SF-2516C rotor (Kubota, Tokyo, Japan), the cells were washed with PBS and resuspended in 1 ml lysis buffer (0.1% Triton X-100, 0.1% RNase A) at 4°C overnight to release the nuclei. Immediately before analysis, propidium iodide (PI; 1 ml, 50 *μ*g ml^−1^ to give a final concentration of 25 *μ*g ml^−1^) was added to the cells. The PI fluorescence of individual nuclei was measured using a FACScan (Becton Dickinson), and the data were analysed using the Cellquest program (Becton Dickinson) and these experiments were repeated twice.

### Indirect immunofluorescence

Cultured cells grown on coverslips in six-well plates were washed twice with PBS and fixed with 100% methanol for 20 min at 4°C. The samples were permeabilised with 1% NP-40 in PBS for 10 min at room temperature, incubated with blocking solution (15% albumin from bovine serum in PBS) for 30 min, and probed with primary antibodies for 1 h at 37°C. The controls in all instances had the primary antibodies omitted. The primary antibody used for immunostaining was rabbit anti-*γ*-tubulin polyclonal antibody. The antibody–antigen complexes were detected with Alexa Fluor 594-conjugated goat anti-rabbit IgG antibody (Molecular Probes, Eugene, OR, USA) for *γ*-tubulin. The samples were washed with TBS buffer and then were counterstained with 4′,6′-diamidino-2-phenylindole (DAPI). These experiments were repeated twice.

### Evaluation of the number of centrosomes

The number of centrosomes in U251MG and D54MG cells was analysed using immunofluorescence staining for *γ*-tubulin on days 2 (48 h) and 5 (120 h) after transfection with siRNA/control or survivin with/without irradiation (4 Gy). The round spots stained with anti-*γ*-tubulin antibody were recognised as centrosomes, and scored per tumour cell. The results are presented as the mean±s.d., with a minimum of 500 cells being scored. Each experiment was repeated at least twice.

### Fluorescence *in situ* hybridisation

Centromeric probes specific for chromosomes 2 and 17 (CEP2-Spectrum Orange, CEP17-Spectrum Green; Vysis Inc., Downers Grove, IL, USA) were used for fluorescence *in situ* hybridisation (FISH) analysis. Fluorescence *in situ* hybridisation was performed as described previously (Kawamura *et al*, 2004). Briefly, the cells on microscope slides were fixed for 5 min each in three changes of freshly prepared methanol–acetic acid (3 : 1) fixative. The cells were denatured at 73°C for 5 min, and then hybridisation was performed at 37°C overnight. The coverslips were removed, and the slides were washed three times (10 min each) in 50% formamide with 2 × saline sodium citrate buffer (SSC) at 45°C, twice in 2 × SSC at 45°C, and then for 5 min at 25°C in 2 × SSC with 0.1% NP-40. The slides were then rinsed in 2 × SSC and counterstained with DAPI.

Chromosome instability in U251MG and D54MG cells was analysed by FISH using fluorescent probes for chromosomes 2 and 17 on days 2 (48 h) and 5 (120 h) after transfection with siRNA/control or survivin. Each centromeric copy number was scored for more than 200 tumour cells. Each experiment was repeated at least twice.

### Detection of apoptosis

Radiation-induced apoptosis was morphologically assessed by TUNEL assay using the *In Situ* Apoptosis Detection Kit (catalogue MK500; Takara Bio Inc., Ootsu, Japan). Cultured cells grown on coverslips in six-well plates were washed twice with PBS and fixed with 4% formaldehyde. Thereafter, they were stained according to the manufacturer's instructions, and photographs were taken under × 200 magnification using a Nikon OPTIPHOT-2 fluorescence microscope. The apoptotic index (AI) was defined as the percentage of TdT-mediated dUTP-biotin nick end labeling (TUNEL)-positive cells in a × 200 magnified field, and the AI values of the experimental groups were compared. Two different people blinded to the treatment counted the positive cells in three microscopic fields on one slide from each specimen and there were no significant differences between the counts that they obtained. Each experiment was repeated at least twice.

### Statistical analysis

Statistical analyses were performed by Student's *t-*test using the SPSS 11.5 software package for Windows (SPSS INC., Chicago, IL, USA). A *P*-value of <0.05 was considered to indicate statistical significance.

## RESULTS

### Suppression of survivin expression

**Figure 1:**
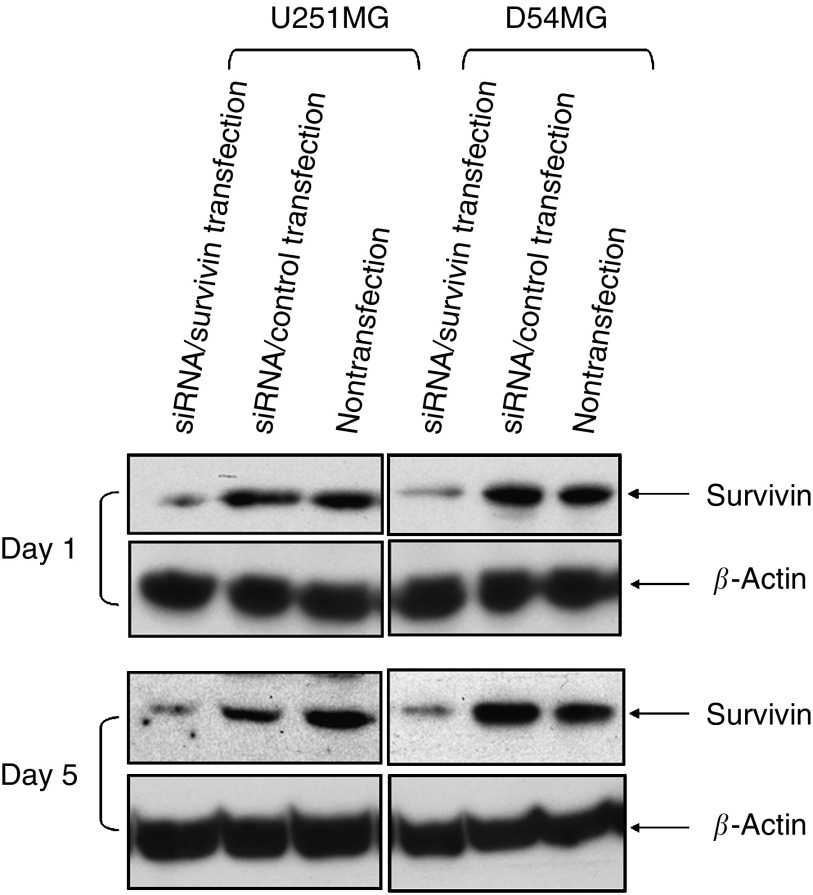
Western blot analyses of the survivin expression in U251MG and D54MG cells at 24 and 120 h (on days 1 and 5) after either transfection with siRNA/survivin, a control or nontransfection. *β*-Actin serves as an internal loading control.

U251MG and D54MG are well-known glioma cell lines that we have previously used to analyse the effect of the p53 status on radiosensitivity (Hama *et al*, 2003). We first used a western blot analysis to examine the effect of siRNA/survivin on U251MG and D54MG cells (Figure 1). Survivin protein expression was observed in both nontransfected and siRNA/control-transfected U251MG and D54MG cells but markedly suppressed in siRNA/survivin-transfected cells. This effect of siRNA transfection using the HVJ Envelope VECTOR KIT was detectable from 24 h until at least 120 h after the transfection as previously described (Watanabe *et al*, 2004). Therefore, we confirmed that siRNA/survivin effectively suppresses the survivin protein level in both cell lines.

### Effect of survivin suppression on radiation-induced cell death

**Figure 2:**
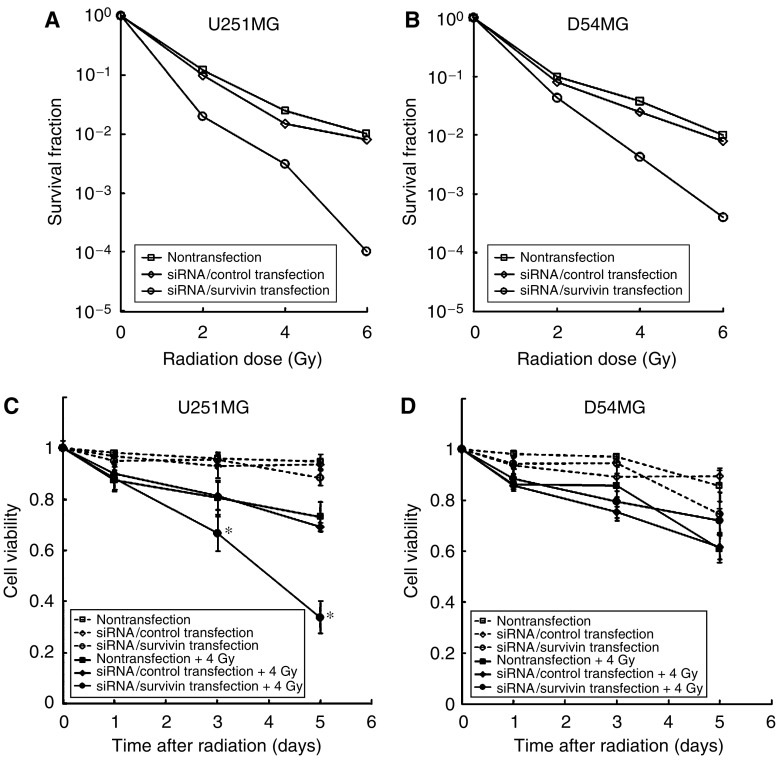
Effect of siRNA/survivin transfection on cell survival or cell viability in U251MG and D54MG cells with/without irradiation. (**A**) U251MG and (**B**) D54MG transfected with either the siRNA/control or survivin with irradiation show the cell survival as determined by clonogenic assays. (**C**) U251MG (p53, mutant type) and (**D**) D54MG (p53, wild-type) transfected with either the siRNA/control or survivin with/without irradiation show cell viability determined using the trypan blue exclusion assay. The results of cell viability assays are shown as the mean and standard deviation of three wells. Statistical significance: ^*^P<0.05, in comparison to siRNA/control-transfected and -irradiated cells.

We examined the effect of survivin suppression on radiosensitivity using a clonogenic survival assay (14 days after irradiation). As shown in Figure 2A and B, the survival rates of both U251MG and D54MG cells were markedly reduced by siRNA/survivin transfection. However, this marked sensitisation was not observed for the siRNA/control-transfected cells.

To examine the cell viability after survivin suppression with or without irradiation, we next examined the effect of survivin suppression on radiosensitivity in U251MG and D54MG cells using a trypan blue exclusion assay for 5 days (120 h) after irradiation (Figure 2C and D). In the preliminary experiments, we compared the cell-killing effect on the radiosensitivity after survivin inhibition between various dose range of irradiation (2, 4, and 6 Gy) using trypan blue exclusion test. The effect of survivin inhibition on the radiosensitivity was little on 2 Gy (U251MG: 71%, D54MG: 82%), and enhanced on 4 Gy (U251MG: 33%, D54MG: 69%). Under the 6 Gy irradiation conditions, the cell-killing effect was maximum (U251MG: 17%, D54MG: 53%), while the number of residual cells was small and such cells were also quite fragile, and almost all the cells peeled off from coverslips during other experiment procedures (evaluation of the number of centrosomes and fluorescence *in situ* hybridisation) and thus were not reproducible (data not shown). We therefore chose an irradiation dose of 4 Gy in the following experiments. On days 3 (72 h) and 5 (120 h) after irradiation, the viability of siRNA/survivin-transfected U251MG cells decreased significantly in comparison to the siRNA/control-transfected cells (*P*=0.005 on day 3, and *P*<0.001 on day 5). In contrast, D54MG cells with siRNA/survivin did not exhibit a significant decrease in viability in comparison to the siRNA/control. Therefore, the clonogenic assay indicated that siRNA/survivin transfection increases radiosensitivity regardless of the p53 status, however, trypan blue exclusion assay for 5 days revealed differences between the two cell lines.

### Effect of survivin siRNA transfection with/without irradiation on the cell cycle

**Figure 3:**
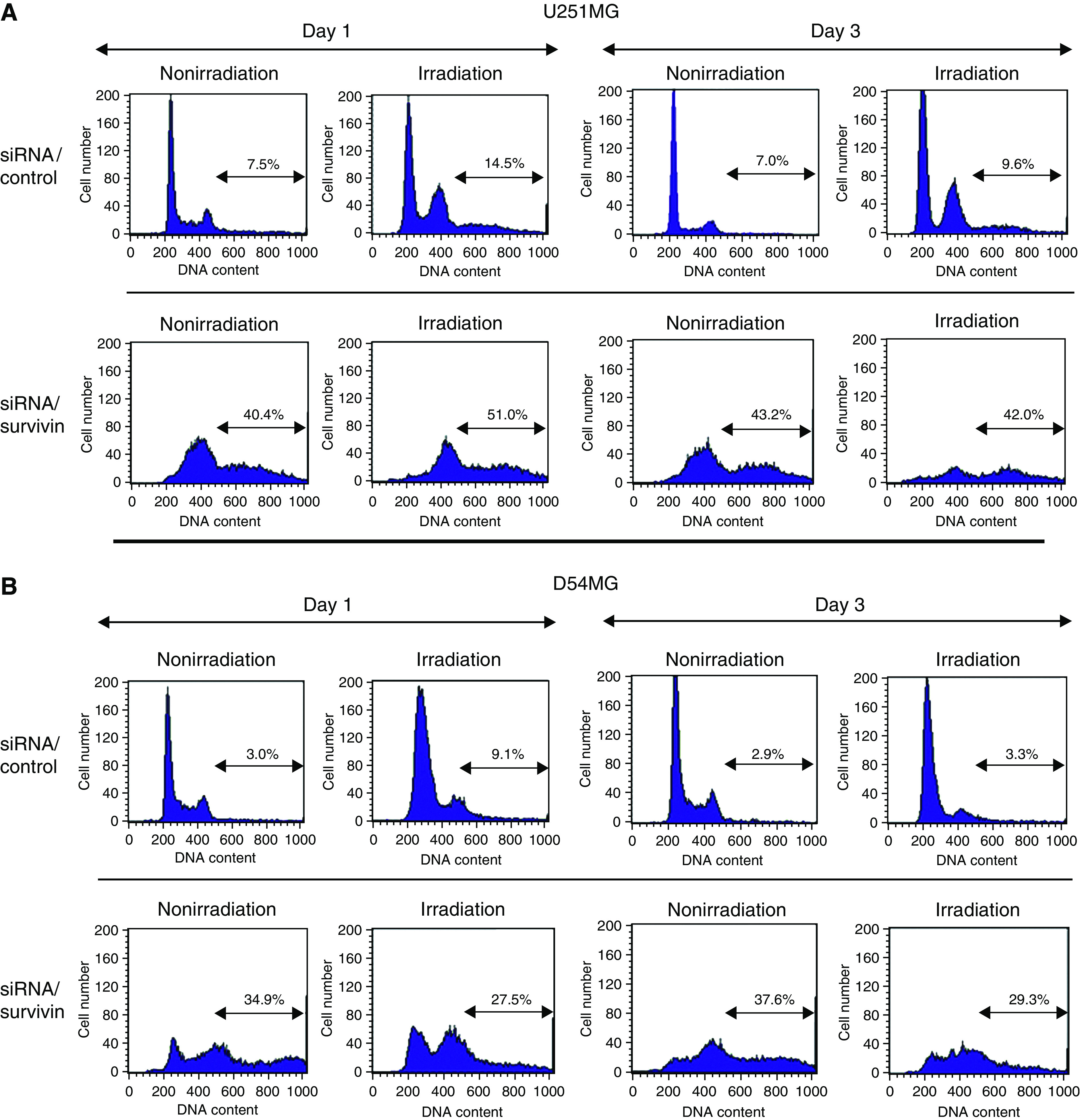
Effect of siRNA/survivin or control transfection on the cell cycle of U251MG and D54MG cells with/without irradiation on days 1 (24 h) and 3 (72 h). The cell-cycle status was analysed by flow cytometry. U251MG (**A**) and D54MG cells (**B**), nonirradiated or irradiated at a dose of 4 Gy.

We used flow cytometry to evaluate the short-term (5 days) cell-cycle effects of transfection with siRNA/control or survivin with/without irradiation (4 Gy). Figure 3 shows the cell-cycle histogram on days 1 (24 h) and 3 (72 h). The data for day 5 (120 h) were almost the same as those for day 3 (data not shown). In U251MG cells (Figure 3A), DNA aneuploidy was markedly developed from day 1 and continued until 5 days after siRNA/survivin transfection in comparison to siRNA/control transfection with/without irradiation (4 Gy). Similarly, in D54MG cells (Figure 3B), siRNA/survivin-transfected cells showed a marked increase in DNA aneuploidy with/without irradiation. However, the degree of DNA aneuploidy in U251MG was markedly higher than that in D54MG cells. Furthermore, the proportion of aneuploid cells of U251MG with siRNA/survivin transfection and irradiation slightly decreased from days 1 to 3, while that of D54MG did not. These results demonstrate that siRNA/survivin transfection with/without irradiation therefore resulted in the development of DNA aneuploidy, the degree of which was different in U251MG and D54MG cells.

### The number of centrosomes in U251MG and D54MG cells

**Figure 4:**
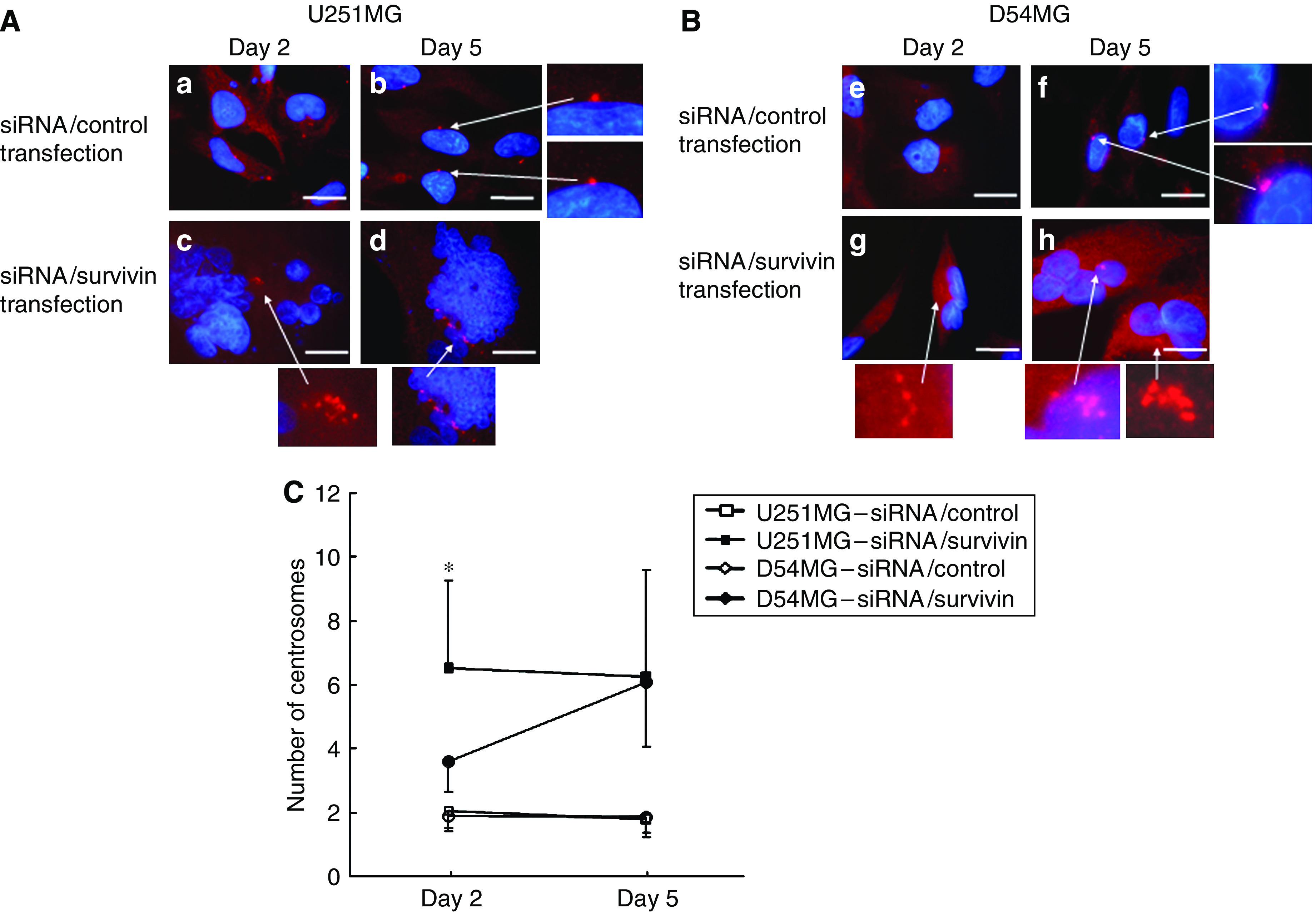
An immunofluorescent analysis of U251MG (**A**) and D54MG (**B**) cells on days 2 (48 h) and 5 (120 h) after transfection with siRNA. Cells treated with siRNA/control or survivin were immunostained for centrosomes (red dot) using anti-*γ*-tubulin and counterstained for DNA with 4′,6′-diamidino-2-phenylindole (blue). (**A**) (**a**, **b**) U251MG cells treated with siRNA/control, (**c**, **d**) U251MG cells treated with siRNA/survivin. (**B**) (**e**, **f**) D54MG cells treated with siRNA/control, (**g, h**) D54MG cells treated with siRNA/control. (**C**) The number of centrosomes in U251MG and D54MG cells transfected with siRNA/control or survivin on days 2 and 5. The results are presented as the mean±s.d. with a minimum of 500 cells being scored. ^*^P<0.001 compared with siRNA/survivin-transfected D54MG cells on day 2. Scale bar, 10 *μ*m.

We next focused on the number of centrosomes after survivin suppression. Recently, a number of studies have shown centrosome amplification to be a contributing factor to CIN in tumour cells (D'Assoro *et al*, 2002; Doxsey, 2002; Kawamura *et al*, 2004). Therefore, centrosome amplification is considered to be a hallmark for CIN. We evaluated the number of centrosomes using an immunofluorescence analysis. Figure 4 demonstrates that siRNA/survivin-transfected cells showed a marked increase in the number of centrosomes in comparison to the corresponding controls in both cell types. However, the number of centrosomes in U251MG cells transfected with siRNA/survivin (6.53±2.74) was significantly higher than that in D54MG cells (3.62±0.99) on day 2 (*P*<0.001). However, as the centrosome number in D54MG cells gradually increased, the difference in centrosome amplification was no longer significant on day 5 (Figure 4C).

**Figure 5:**
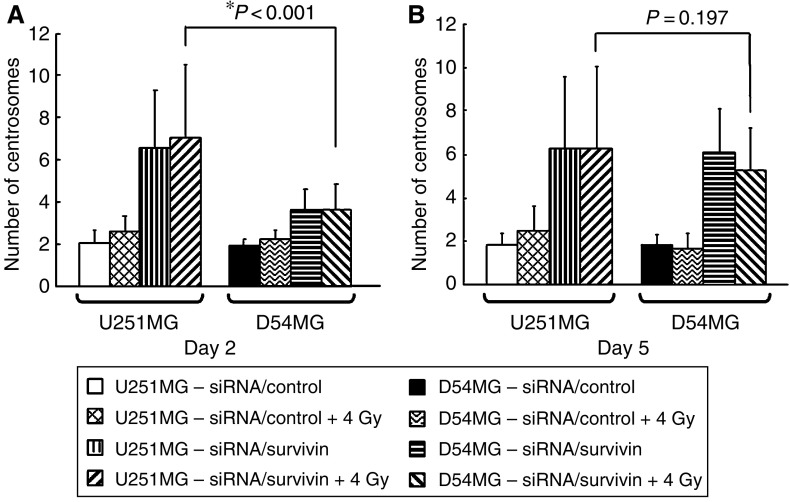
Comparison of the number of centrosomes in U251MG and D54MG cells on days 2 (48 h) and 5 (120 h) after siRNA/survivin or control transfection with/without irradiation. (**A**) The centrosome number on day 2 was significantly higher for U251MG cells with irradiation and transfection with siRNA/survivin than for D54MG cells receiving the same treatment (^*^P<0.001). (**B**) On day 5, there was no statistically significant difference in the centrosome number between irradiated and siRNA/survivin-transfected U251MG cells and D54MG cells under the same conditions (*P*=0.197).

As shown in Figure 5, the number of centrosomes in the siRNA/survivin-transfected and irradiated (4 Gy) cells also showed a marked increase in comparison to the siRNA/control-transfected cells with/without irradiation on day 2 in both cell lines. However, the number of centrosomes in U251MG cells with irradiation and siRNA/survivin transfection (7.02±3.52) was significantly higher than that in D54MG cells (3.60±1.23; *P*<0.001). However, no significant difference in centrosome amplification between the cell lines with irradiation and siRNA/survivin transfection was observed on day 5 (U251MG: 6.30±3.72; D54MG: 5.25±1.93). These results indicate that siRNA/survivin transfection enhances the increase in the number of centrosomes in both cell lines with/without irradiation, but the increase in U251MG cells was more evident and occurred earlier than in D54MG cells.

### Effect of survivin siRNA transfection on chromosome instability

**Figure 6:**
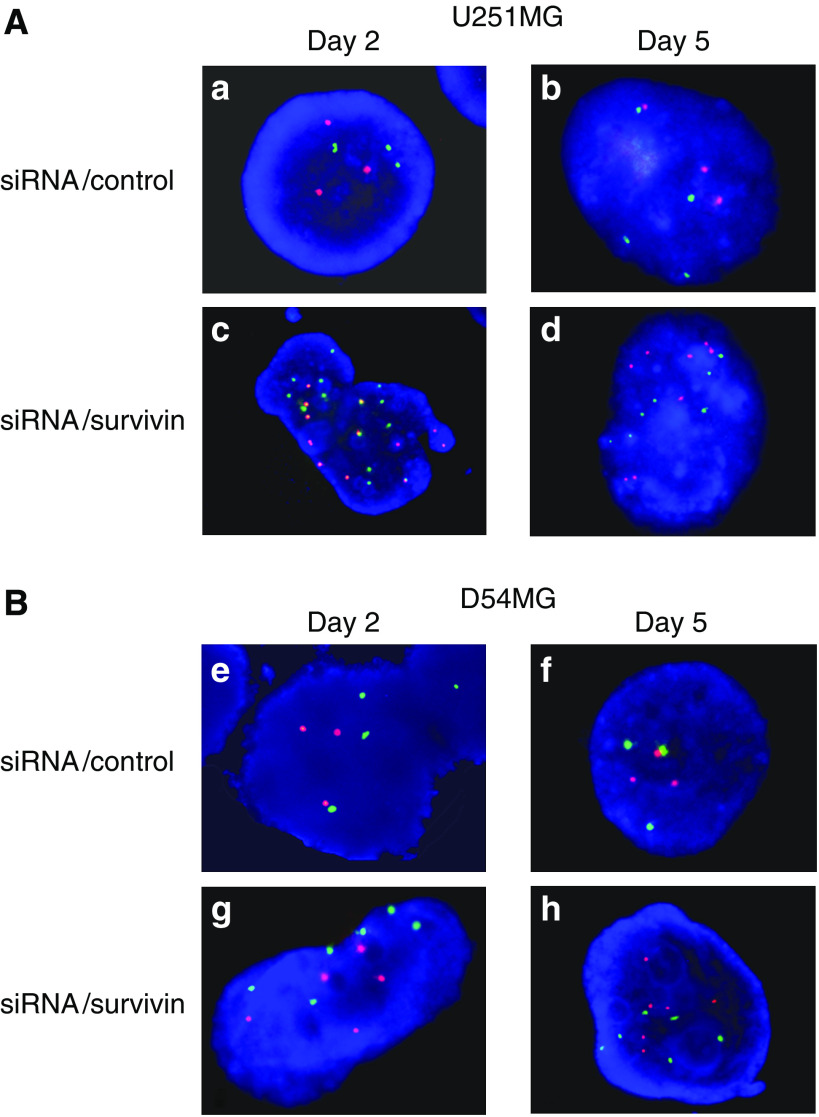
A FISH analysis of U251MG (**A**) and D54MG (**B**) cells on days 2 (48 h) and 5 (120 h) after transfection with siRNA. The cells treated with siRNA/control or survivin were examined by FISH using fluorescent probes for chromosomes 2 (red) and 17 (green). (**A**) (**a, b**) U251MG cells treated with siRNA/control, (**c, d**) U251MG cells treated with siRNA/survivin. (**B**) (**e, f**) D54MG cells treated with siRNA/control, (**g, h**) D54MG cells treated with siRNA/control.

**Figure 7:**
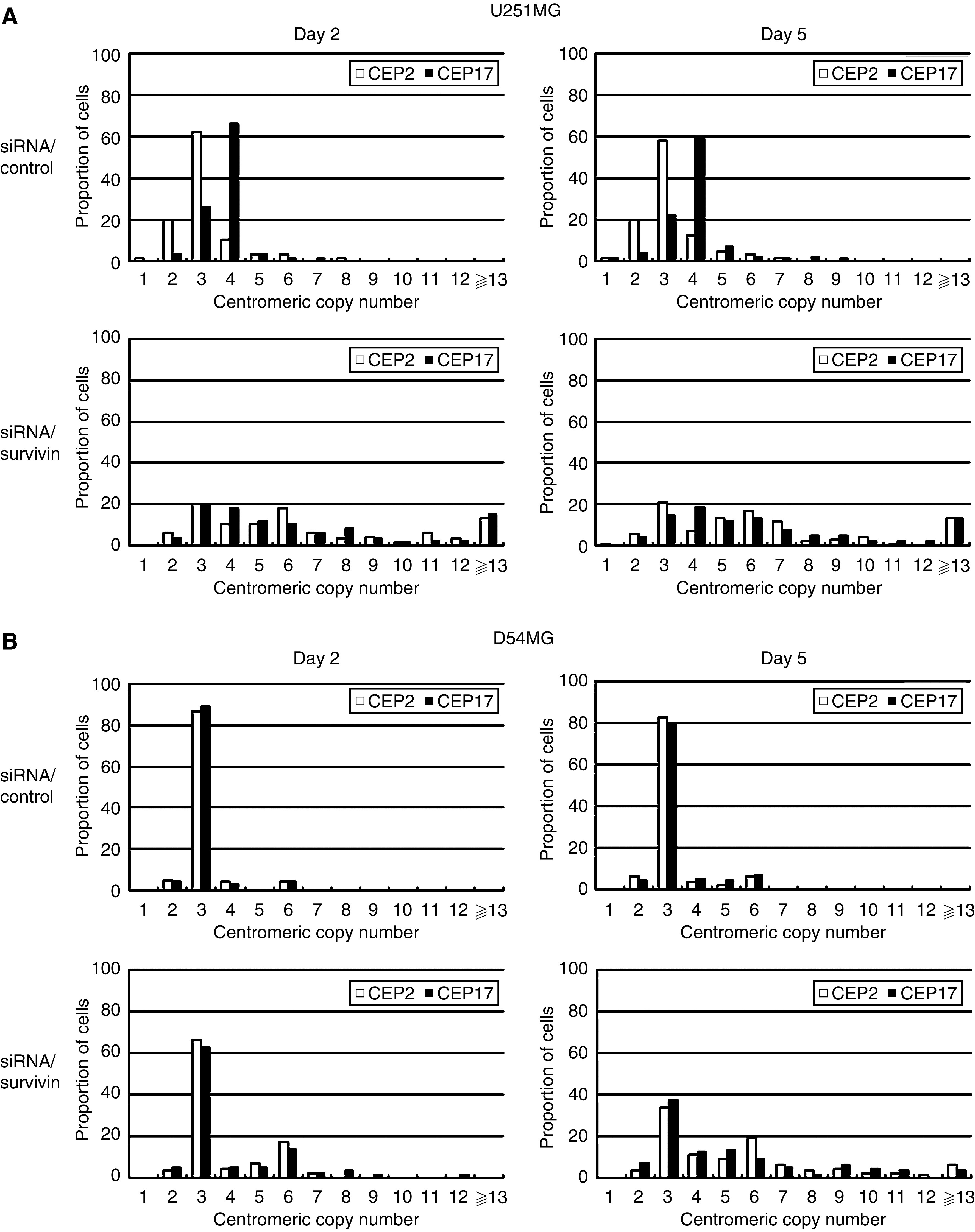
An analysis of chromosome instability in U251MG (**A**) and D54MG (**B**) cells. The chromosome instability was analysed by FISH using fluorescent probes for chromosomes 2 and 17 on days 2 (48 h) and 5 (120 h) after transfection with siRNA/control or survivin. Each centromeric copy number was scored for more than 200 tumour cells.

To confirm whether the centrosome amplification in this study indicated CIN or not, we analysed the centromeric copy number by FISH using fluorescent probes 2 and 17 on days 2 and 5. On day 2, the FISH analysis of D54MG cells revealed that >88% of cells showed three spots for chromosome 2 and 17 probes, thus indicating that these chromosomes are stable in D54MG cells (Figures 6B and 7B). In contrast, many U251MG cells showed three spots for chromosome 2 (62%) and four spots for chromosome 17 (66%), but the chromosome numbers were less stable than those of D54MG cells (Figures 6A and 7A). After the transfection of siRNA/survivin, D54 MG cells showed various numbers of spots and the percentage of three spots for chromosomes 2 and 17 decreased, thus indicating a small increase in chromosome number instability. However, U251MG cells with siRNA/survivin transfection showed a marked increase in the number and multiplicity of spots for chromosomes 2 and 17 in comparison to those with siRNA/control transfection, suggesting that CIN was induced by siRNA/survivin transfection. On day 5, U251MG cells with siRNA/survivin transfection showed a large increase in the number and multiplicity of spots, as did D54MG cells with siRNA/survivin transfection. These results confirmed the centrosome amplification observed in this study to reflect CIN, thereby suggesting that number of centrosome correlated with the degree of CIN. Although siRNA/survivin transfection increased the degree of CIN in both cell lines, the instability was more evident and occurred earlier in U251MG cells than in D54MG cells. Consequently, the degree of CIN is thus considered to correlate with the number of centrosomes after survivin/siRNA transfection in both cell lines.

### Apoptotic analysis

**Figure 8:**
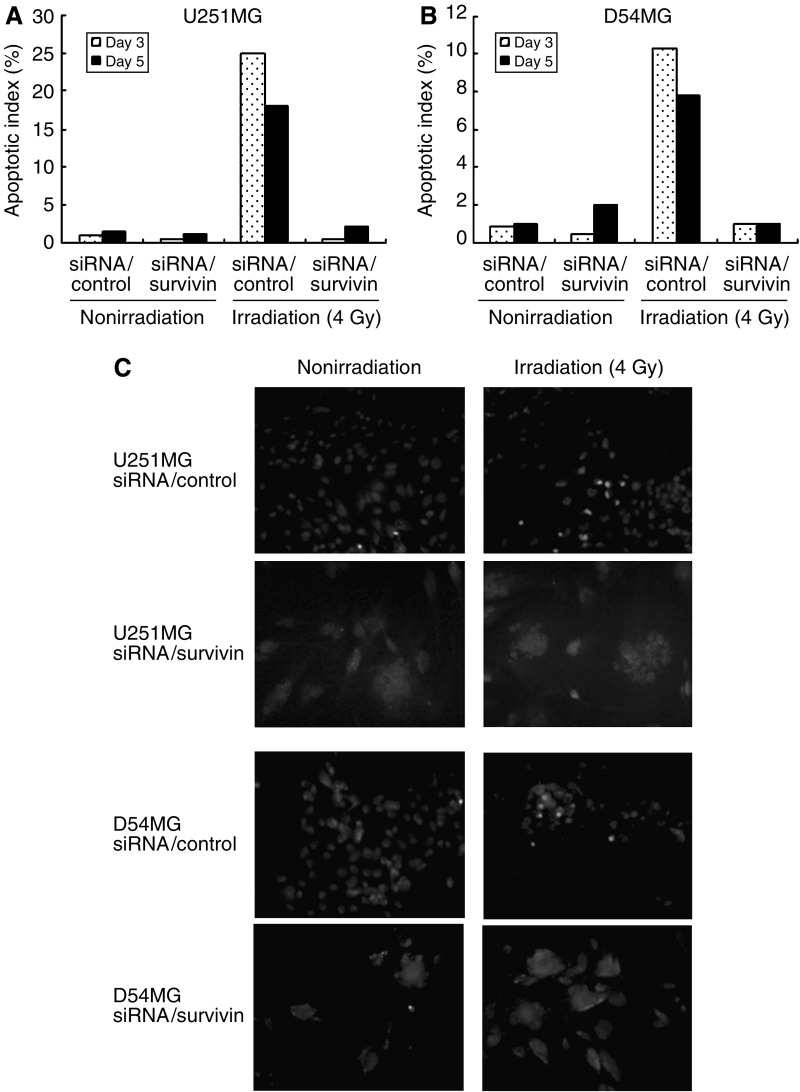
Apoptosis was assessed by TUNEL assay. The TUNEL assay was used for the morphological detection of any apoptotic changes. U251MG or D54MG cells with siRNA/control or survivin transfection, nonirradiated or irradiated at a dose of 4 Gy, were cultured for 3 (72 h) or 5 days (120 h), and were stained according to the manufacturer's instructions after fixation with 4% formaldehyde. The AI was defined as the percentage of TUNEL-positive U251 (**A**) or D54MG cells (**B**). (**C**) TUNEL signals were detected by fluorescence microscopy in the nuclei of some irradiated and siRNA/control-transfected U251MG cells.

To determine whether the death of siRNA/survivin-transfected U251MG and D54MG cells after irradiation was due to apoptosis, we performed a TUNEL assay and measured the level of apoptosis based on the TUNEL positivity on days 3 (72 h) and 5 (120 h) either with or without irradiation. Figure 8A and B show the percentage of TUNEL-positive cells. Irradiated cells that were transfected with siRNA/control had a higher proportion of TUNEL-positive cells than the corresponding nonirradiated cells in both the cell lines on days 3 and 5. However, irradiated cells that were transfected with siRNA/survivin had the same proportion of TUNEL-positive cells as the corresponding nonirradiated cells in both the cell lines on days 3 and 5. As shown in Figure 8C, the irradiated siRNA/control-transfected cells were identified as scattered clusters of fluorescent staining, suggesting the death of these cells to be due to apoptosis in both the cell lines. In contrast, siRNA/survivin-transfected cells did not emit fluorescent signals even after irradiation in both the cell lines. Therefore, the cell death of the siRNA/survivin-transfected cells following irradiation is not considered to be due to apoptosis.

## DISCUSSION

The present results demonstrated that the radiosensitisation differed with p53 status as U251MG cells (p53-mt) quickly developed extreme CIN (centrosome number ⩾5–7) and enhanced radiosensitivity, while CIN and radiosensitivity increased more gradually in D54MG cells (p53-wt). It is surprising that the radiosensitivity did not increase when the degree of CIN was mild (centrosome number ⩽3–4), while radiosensitivity markedly increased in line with the elevated degree of CIN (centrosome number ⩾5–7) after the suppression of survivin.

To standardise the genetic background of the two cell lines used in this study (U251MG and D54MG), other than the p53 gene, we inhibited wild-type p53 of D54MG cells (p53-wt) using siRNA/p53 transfection, and then examined both centrosome amplification (Supplementary Figure 1) and FISH (Supplementary Figure 2); we demonstrated that centrosome amplification and chromosome number instability in p53-inhibited D54MG cells developed earlier and were more extreme than in un-inhibited D54MG cells. D54MG cells with siRNA/p53 transfection showed the same behaviour as U251MG cells after survivin inhibition, thus proving that the degree of centrosome amplification and CIN induced by survivin inhibition depends on the cells' p53 status.

The question arose as to why radiosensitivity was different depending on the p53 status after survivin inhibition. The effect of survivin inhibition on the radiosensitivity remains controversial: some studies have reported the effect of survivin inhibition (using siRNA transfection) on radiosensitivity (estimated with colony assay) to be mediated by the p53 status, while others have reported the opposite (Kappler *et al*, 2005; Rodel *et al*, 2005). However, our present data also demonstrated the centrosome aberration to differ in line with the p53 status after survivin inhibition, thus suggesting that centrosome aberration might demonstrate the interrelationship between the radiosensitising effect and the p53 pathway after survivin inhibition. To maintain chromosomal stability, the postmitotic G1 tetraploid checkpoint blocks the cell cycle of tetraploid cells that escape from G2, and there is a mitotic checkpoint at the G1 phase (Andreassen *et al*, 2003), and this G1 tetraploid checkpoint is thought to be p53-dependent (Andreassen *et al*, 2003). The cells with nonfunctional p53 protein are allowed to go through the rounds of cell division even if their chromosome segregation is incomplete. Therefore, the glioma cell line with wt-p53 might partially block a further proliferation of aneuploid cells and centrosome number leading to CIN. Therefore, our data might demonstrate that centrosome amplification and radiosensitivity were immediately and markedly enhanced in p53-mut cells, but only gradually enhanced in p53-wt cells after survivin inhibition.

Generally, the destabilisation of chromosomes promoted by centrosome amplification aids the acquisition of further malignant phenotypes while promoting tumour progression (Kawamura *et al*, 2004; Fukasawa, 2005). Centrosome amplification frequently occurs in almost all types of cancer, and it is considered to be the major factor contributing to CIN in cancer cells (D'Assoro *et al*, 2002; Kawamura *et al*, 2004; Fukasawa, 2005). Chromosome instability is clinically malignant and thought to be a worsening prognostic factor (Yamamoto *et al*, 2004). However, only a few studies have examined the association between centrosome aberration leading to CIN and radiosensitivity, and the findings of such studies remain controversial. Limoli *et al* (2001) reported that the process of chromosomal breakage and recombination that accompanies CIN might provide some selective pressure for radioresistant variants. On the other hand, Sato *et al* (2000) showed that centrosome overduplication may be a critical event leading to mitotic failure and subsequent cell death following exposure to ionising radiation. Our data showed that survivin inhibition led to radiosensitisation proportional to both the centrosome amplification and chromosome number instability (=degree of CIN). Previous studies examining the association between CIN and radiosensitivity have focused on the CIN inherent in cancer cells (Sato *et al*, 2000; Limoli *et al*, 2001) and thus, the degree of CIN was not as advanced as in our study. Therefore, the degree of CIN might determine the radiosensitisation; the induction of advanced CIN (centrosome number⩾5–7) using survivin inhibition might thus cause an enhancement of radiosensitisation. Therefore, survivin could be an attractive therapeutic target for overcoming the radioresistance of malignant gliomas.Recently, McLaughlin *et al* (2006) reported that epigallocatechin-3-gallate, a green tea-derived anticancer molecule, overcome survivin overexpression-induced radioresistance via downregulation of Rho A. Moreover, guggulsterone, derived from *Commiphora mukul* and used to treat obesity and diabetes, decreases the expression of NF-*κ*B, thus leading to a downregulation of survivin while also enhancing chemo-radiosensitivity (Shishodia and Aggarwal, 2004). These treatments were based on natural products that could regulate both survivin and the survivin-related pathway, thereby making it possible to overcome the radioresistance of malignant gliomas.

We have shown that the radiation-induced cell death following survivin inhibition was nonapoptosis. The study of Temme *et al* (2003) was consistent with our present findings namely that the survivin inhibition induced aberrant cytokinesis, while not inducing apoptosis even after the administration of the DNA-damaging reagents. Furthermore, Silke and Vaux (2001) analysed the structure and function of various member of inhibitor of apoptosis (IAP) family and suggested that survivin does not possess any residues that bind to caspase-3 unlike the other members of IAP family and it also does not play any direct role in apoptosis in mammals, however, it is essential for mitosis. From these observations, it was suggested that the essential function of survivin was therefore not the inhibition of apoptosis but the regulation of cytokinesis including chromosomal segregation. The cell death, which was associated with the gross abnormalities of chromosomal segregation, as shown in our present study, was observed in some cancer cells following the failure of complete mitosis after DNA damage. This type of cell death is called ‘mitotic catastrophe’ or ‘mitotic cell death’. Mitotic cell death was nonapoptotic and shown to correlate with CIN. The data of flow cytometry (Figure 3) indicate that the accumulation of aneuploid cells in siRNA/survivin-transfected and -irradiated U251MG cells decreased from days 1 to 3, in comparison to the siRNA/survivin-transfected and -irradiated D54MG cells, and the trypan blue exclusion test showed that U251MG cells died between days 3 and 5 but D54MG did not show the same findings after the combined treatment with siRNA/survivin and irradiation. This finding is consistent with mitotic cell death. Accumulated aneuploid cells gradually induced cell death, while decreasing the proportion of aneuploid cells after irradiation. Survivin and p53 are believed to play an important role in the coupling of abnormal mitotic progression to the onset of mitotic catastrophe (Castedo *et al*, 2004). Our findings therefore suggest that advanced CIN, such as that observed in a survivin-inhibited state, would therefore reduce the viability after genotoxic stress, including irradiation, thus leading to mitotic cell death and resulting in enhanced radiosensitivity.

In conclusion, we have herein shown that a downregulation of survivin enhanced the radiosensitivity accompanied by centrosome amplification in human glioma cells. Although CIN reflects histological malignancy, advanced CIN (centrosome amplification) thus demonstrates a greater radiosensitivity and therapeutic value in proportion to the increase in the degree of CIN. Therefore, this study provides information regarding the potential curative effect of CIN, and thus paying proper attention to the degree of CIN (centrosome number) may thus be of crucial importance in improving radiation therapy for the treatment of human glioma patients.
