# Supplementary figures and images for: Centrosome amplification induced by survivin suppression enhances both chromosome instability and radiosensitivity in glioma cells
Source: Br J Cancer. 2008 Jan 15;98(2):345–55. doi: 10.1038/sj.bjc.6604160 (PMC2361434; doi:10.1038/sj.bjc.6604160)

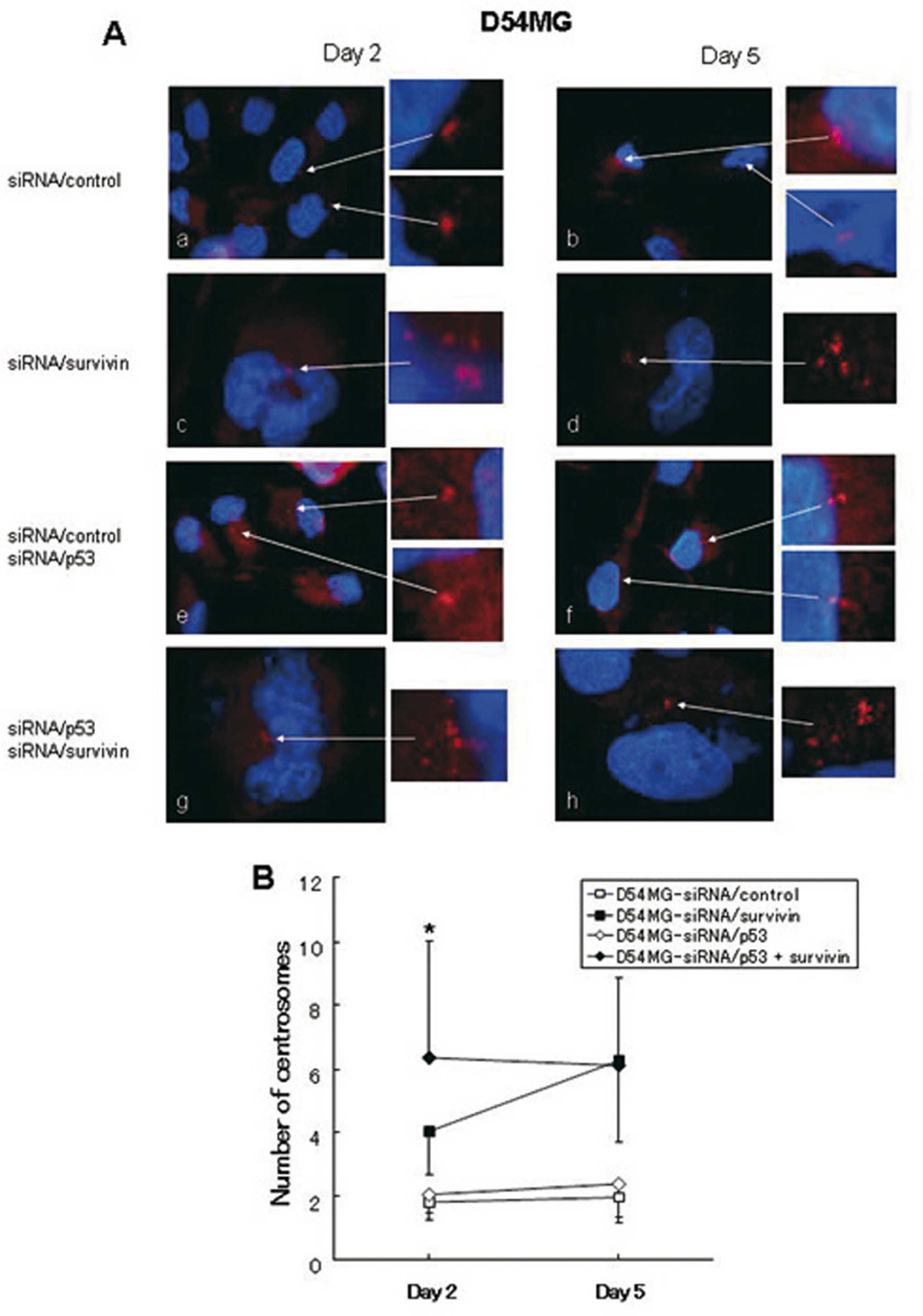

Supplement: Supplementary Figure 1 [file 6604160x1.tif]

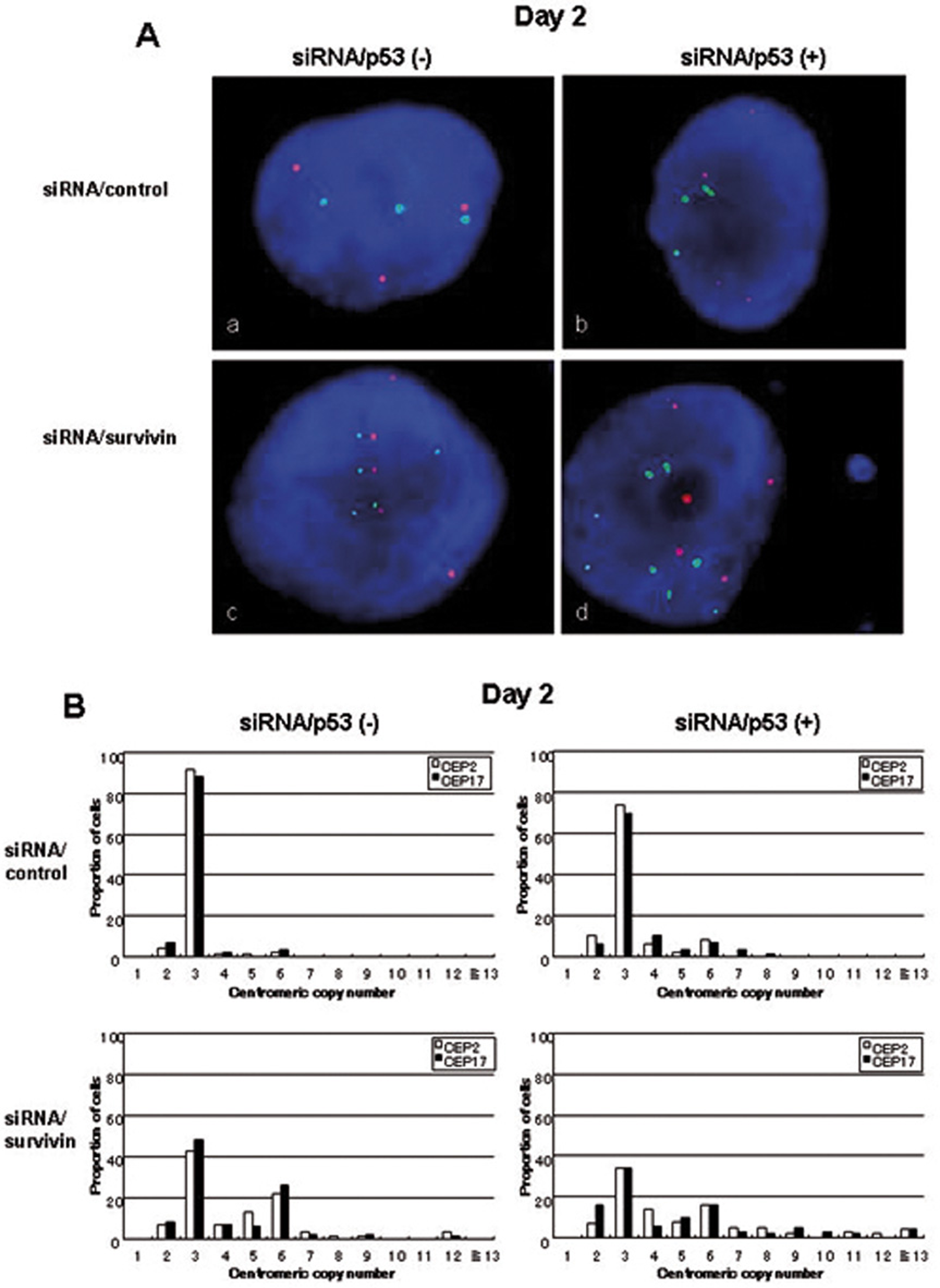

Supplement: Supplementary Figure 2 [file 6604160x2.tif]
