# Supplementary material for: Centrosome amplification induced by survivin suppression enhances both chromosome instability and radiosensitivity in glioma cells
Source: Br J Cancer. 2008 Jan 15;98(2):345–55. doi: 10.1038/sj.bjc.6604160 (PMC2361434; doi:10.1038/sj.bjc.6604160)
Supplement: Supplementary Data [file 6604160x3.doc]

**Supplementary Figure 1**

An immunofluorescent analysis of D54MG cells with/without siRNA/p53 on days 2 (48 h) and 5 (120 h) after transfection with or without siRNA/survivin. The cells treated with siRNA were immunostained for centrosomes (red dot) using anti-γ-tubulin and counterstained for DNA with 4′,6′-diamidino-2-phenylindole (blue). **(A) (a, b)** D54MG cells treated with siRNA/control, **(c, d)** D54MG cells treated with siRNA/survivin, **(e, f)** D54MG cells treated with siRNA/control and p53, **(g, h)** D54MG cells treated with siRNA/p53 and survivin **(B)** The number of centrosomes in D54MG cells with/without siRNA/p53 on days 2 (48 h) and 5 (120 h) after transfection with or without siRNA/survivin. The results are presented as the mean ± SD with a minimum of 500 cells being scored. *: *P* < 0.001 compared with siRNA/survivin transfected-D54MG cells on day 2.

**Supplementary Figure 2**

**(A) A** FISH analysis of D54MG cells with/without siRNA/p53 on day 2 (48 h) after transfection with or without siRNA/survivin. Cells treated with siRNA were examined by FISH using fluorescent probes for chromosomes 2 (red) and 17 (green). **(A) (a)** D54MG cells treated with siRNA/control, **(b)** D54MG cells treated with siRNA/survivin, **(c)** D54MG cells treated with siRNA/control and p53, **(d)** D54MG cells treated with siRNA/p53 and survivin. (**B**) An analysis of chromosome instability in D54MG cells with/without siRNA/p53 on day 2 (48 h) after transfection with or without siRNA/survivin. The chromosome instability was analyzed by FISH using fluorescent probe for chromosomes 2 and 17 on day 2 (48 h) after transfection with siRNA/control or survivin. Each centromeric copy number was scored per more than 200 tumor cells.

**Supplementary Figure to referee**

Effect of siRNA/survivin transfection on cell viability in U251MG and D54MG cells between various doses range irradiation (2, 4 and 6 Gy) using trypan-blue exclusion test. (**A**) U251MG and D54MG cells transfected with siRNA/control or survivin after various doses range irradiation show cell viability on day 3. (**B**) U251MG and D54MG cells transfected with siRNA/control or survivin after various doses range irradiation show cell viability on day 5. The results of cell viability assays are shown as the mean and standard deviation of three wells.
